# Synergistic Effects of Polyphenols and Methylxanthines with Leucine on AMPK/Sirtuin-Mediated Metabolism in Muscle Cells and Adipocytes

**DOI:** 10.1371/journal.pone.0089166

**Published:** 2014-02-14

**Authors:** Antje Bruckbauer, Michael B. Zemel

**Affiliations:** Research and Development, NuSirt Sciences Incorporated, Knoxville, Tennessee, United States of America; Pennington Biomedical Research Center, United States of America

## Abstract

The AMPK-Sirt1 pathway is an important regulator of energy metabolism and therefore a potential target for prevention and therapy of metabolic diseases. We recently demonstrated leucine and its metabolite β-hydroxy-β-methylbutyrate (HMB) to synergize with low-dose resveratrol (200 nM) to activate sirtuin signaling and stimulate energy metabolism. Here we show that leucine exerts a direct effect on Sirt1 kinetics, reducing its Km for NAD^+^ by >50% and enabling low doses of resveratrol to further activate the enzyme (p = 0.012). To test which structure elements of resveratrol are necessary for synergy, we assessed potential synergy of structurally similar and dissimilar polyphenols as well as other compounds converging on the same pathways with leucine using fatty acid oxidation (FAO) as screening tool. Dose-response curves for FAO were constructed and the highest non-effective dose (typically 1–10 nM) was used with either leucine (0.5 mM) or HMB (5 µM) to treat adipocytes and myotubes for 24 h. Significant synergy was detected for stilbenes with FAO increase in adipocytes by 60–70% (p<0.05) and in myotubes >2000% (p<0.01). Sirt1 and AMPK activities were stimulated by ∼65% (p<0.001) and ∼50% (p<0.03), respectively. Similarly, hydroxycinnamic acids and derivatives (chlorogenic, cinnamic, and ferulic acids) combined with leucine/HMB increased FAO (300–1300%, p<0.01), AMPK activity (50–150%, p<0.01), and Sirt1 activity (∼70%, p<0.001). In contrast, more complex polyphenol structures, such as ellagic acid and epigallocatechin gallate required higher concentrations (>1 µM) and exhibited little or no synergy. Thus, the six-carbon ring structure bound to a carboxylic group seems to be a necessary element for leucine/HMB synergy with other stilbenes and hydroxycinnamic acids to stimulate AMPK/Sirt1 dependent FAO; these effects occur at concentrations that produce no independent effects and are readily achievable via oral administration.

## Introduction

AMP-activated protein kinase (AMPK) and the sirtuins Sirt1 and Sirt3 are well-known key sensors of energy status and regulators of glucose and lipid metabolism [1]–[3]. They work in a finely tuned network with the peroxisome proliferator activated receptor γ co-activator 1α (PGC-1α) to regulate mitochondrial proliferation and metabolism and energy expenditure [4], [5]. Accordingly, this network appears to be a strong target for prevention and control of metabolic diseases such as obesity and diabetes.

The polyphenol resveratrol (Resv), found in the skin of red grapes and other fruits, has been reported to be a Sirt1 activator, mimicking the effects of caloric restriction on life span, oxidative and inflammatory stress, as well as improving insulin sensitivity and reducing adiposity [6], [7]. However, Sirt1 activation by Resv has been suggested by some to be a measurement artifact, as direct Sirt1 activation demonstrated with a fluorophore-linked enzyme activity assay (Fleur-de-Lys assay) was dependent on the presence of the fluorophore [8], [9]. In contrast, recent data indicates that, depending on the substrate, the fluorophore was substituting for endogenously present hydrophobic amino acids such as leucine to link Resv with the substrate to activate Sirt1 [10]. In addition, there is evidence for an indirect Sirt1 activation mediated by inhibiting cAMP phosphodiesterase, which results in upregulation of AMPK and a subsequent increase in NAD^+^ levels [11]. However, this was shown to be the case only at high concentrations (50 µM) that are not achieved *in *vivo, while lower concentrations lead to direct Sirt1 activation [12]. Thus, these different modes of action may explain reports of Resv’s dose- and time- dependent effects, which lead to different outcomes in cell and animal studies. However, studies in humans are very limited and results from cell and animals studies are not readily translated, since, due to the low bioavailability of Resv, plasma concentrations achieved with oral supplementation are much lower than those used *in vitro*.

We have previously demonstrated that the branched-chain amino acid leucine (Leu), as well as its metabolites β-hydroxy-β-methylbutyrate (HMB) and α-ketoisocaproate (KIC), directly activate recombinant human Sirt1 enzyme by 30 to 100% [13]. In addition, we have shown that Leu or HMB act synergistically with low concentrations of Resv to increase Sirt1 and AMPK activity resulting in improved insulin sensitivity, and increased muscle glucose and palmitate uptake *in vitro* and *in vivo* [14].The concentrations of Leu and Resv used exerted minimal independent effects and can be achieved via oral administration. Notably, these synergistic effects were more pronounced under high glucose media conditions simulating glycemic stress. Thus this combination may play a therapeutic role in obesity and diabetes prevention and management. However, it is not clear whether this synergy with Leu is a unique feature of Resv or can also be extrapolated to other compounds with structural similarity or to compounds converging on the same pathways.

To address these issues, we first assessed the direct effects of Leu on Sirt1 activity and kinetics using both the Fleur-de-Lys assay and a FRET-based assay that is not subject to artifact resulting from fluorophore binding, and evaluated Resv activation of Sirt1in the presence and absence of Leu in both systems. We then assessed synergy between Leu or HMB with polyphenols, selected based on the degree of their chemical structural similarity to Resv. We also evaluated compounds that converge on the same metabolic pathways as activated by Resv/Leu, in order to understand what elements may be a requirement for synergy. Since fatty acid oxidation is a key outcome measure of AMPK/Sirt1 activation, we used palmitate-induced oxygen consumption rate as a sensitive first level of screening for aerobic mitochondrial metabolism.

## Materials and Methods

### Cell Culture

Murine 3T3-L1 pre-adipocytes were incubated at a density of 8000 cells/cm^2^ (10 cm^2^ dish) and grown in the absence of insulin in Dulbecco’s modified Eagle’s medium (DMEM, 25 mM glucose) containing 10% fetal bovine serum (FBS) and antibiotics (1% penicillin-streptomycin)(adipocyte medium) at 37°C in 5% CO_2_ in air. Confluent pre-adipocytes were induced to differentiate with a standard differentiation medium (DM2-L1, Zen-Bio Inc., NC). Pre-adipocytes were maintained in this differentiation medium for 3 days and subsequently cultured in adipocyte medium for further 8 to 10 days to allow at least 90% of cells to reach fully differentiation before treatment. Media was changed every 2–3 days; differentiation was determined microscopically via inclusion of fat droplets.

Murine C2C12 muscle cells were incubated at a density of 8000 cells/cm2 (10 cm^2^ dish) and grown in DMEM containing 10% FBS and antibiotics (adipocyte medium) at 37°C in 5% CO2 in air. Cells were grown to 100% confluence, changed into differentiation medium (DMEM with 2% horse serum and 1% penicillin– streptomycin), and fed with fresh differentiation medium every day until myotubes were fully formed (6 days).

Treatment concentrations for all cell experiments were 200 nM Resv, 5 uM HMB, and 0.5 mM Leu; incubation time was between 4 and 48 h, depending on experiment.

### Fatty Acid Oxidation

Cellular oxygen consumption was measured using a Seahorse Bioscience XF24 analyzer (Seahorse Bioscience, Billerica, MA) in 24-well plates at 37°C, as described by Feige et al [15] with slight modifications. Cells were seeded at 40,000 cells per well, differentiated as described above, treated for 24 hours with the indicated treatments, washed twice with non-buffered carbonate-free pH 7.4 low glucose (2.5 mM) DMEM containing carnitine (0.5 mM), equilibrated with 600 µL of the same media in a non-CO_2_ incubator for 30 minutes, and then inserted into the instrument for 15 minutes of further equilibration. O_2_ consumption was measured in three successive baseline measures at eight-minute intervals prior to injection of palmitate (200 µM final concentration). Post-palmitate-injection measurements were taken over a 4-hour period with cycles consisting of 10 min break and three to five successive measurements of O_2_ consumption.

### Sirt1 Activity (Fleur-de-Lys)

Sirt1 activity was measured by using the Sirt1 Fluorimetric Drug Discovery Kit (BML-AK555, ENZO Life Sciences Inc., Farmingdale, NY, USA). The sensitivity and specifity of this assay kit was tested by Nin et al. [16]. In this assay, Sirt1 activity is assessed by the degree of deacetylation of a standardized substrate containing an acetylated lysine side chain. The substrate utilized is a peptide containing amino acids 379–382 of human p53 (Arg-His-Lys-Lys[Ac]), an established target of Sirt1 activity; Sirt1 activity is directly proportional to the degree of deacetylation of Lys-382. Samples were incubated with peptide substrate (25 µM), and NAD^+^ (500 µM) in a phosphate-buffered saline solution at 37°C on a horizontal shaker for 45 minutes. The reaction was stopped with the addition of 2 mM nicotinamide and a developing solution that binds to the deacetylated lysine to form a fluorophore. Following 10 minutes incubation at 37°C, fluorescence was read in a plate-reading fluorimeter with excitation and emission wavelengths of 360 nm and 450 nm, respectively. Resv (100 mM) served as a Sirt1 activator (positive control) and suramin sodium (25 mM) as a Sirt1 inhibitor (negative control). The endogenous Sirt1 activity in muscle cell and mouse white adipose tissue (WAT) was measured in a modified assay using 5 µl of cell lysate. The lysates were prepared by homogenizing cells in ice-cold RIPA buffer plus protease inhibitor mix (Sigma Aldrich Corp., St. Louis, MO, USA). After 10 min incubation on ice, the homogenate was centrifuged at 14,000 *g* and the supernatant was used for further experiments. Data for endogenous Sirt1 activation were normalized to cellular protein concentration measured via BCA-assay.

### Sirt1 FRET-based Screening Assay Kit (Cayman, # 10010991)

This assay is a fluorescence-based method for screening of Sirt1 inhibitors and activators. It can be used to eliminate false Sirt1 activation found with the coumarin-based substrate as used in the above assay.

First human recombinant Sirt1 enzyme is incubated with the substrate, which is coupled to the fluorophore, and a quencher along with its cosubstrate NAD^+^. The Sirt1 mediated deacetylation sensitizes the substrate such that the developer, which is added in the second step, separates the quencher and fluorophore. The emitted fluorescence can be measured in a plate-reading fluorimeter with excitation and emission wavelengths of 335–345 nm and 440–465 nm, respectively. This assay was modified by diluting NAD^+^ to the indicated concentrations.

### AMPK Activity

AMPK activity in cells was measured via the AMPK Kinase Assay Kit (CycLex Co., Ltd., Nagano, Japan) according to manufacture’s instruction. This assay provides a non-isotopic, sensitive and specific method in form of an ELISA and uses anti-phospho-mouse insulin receptor substrate (IRS)-1 S789 monoclonal antibody and peroxidase coupled anti-mouse IgG antibody as a reporter molecule. The amount of phosphorylated substrate is determined by measuring absorbance at 450 nm. Differentiated cells were incubated with indicated treatments for 24 h. Cells were washed three times with ice-cold phosphate buffered saline (PBS), then lysed in Cell Lysis Buffer for 90 minutes on ice, centrifuged at 3,500 rpm for 15 min at 4°C. Then 10 µl of clear supernatant was used for each assay experiment. Purified recombinant AMPK active enzyme was included as a positive control for phosphorylation. 0.5 mM AICAR was included in some experiments as an AMPK activator. To calculate the relative AMPK activity of the samples, an inhibitor control with Compound C for each sample was included once and inhibitor control absorbance values were subtracted from test sample absorbance values.

### Statistical Analysis

All data were expressed as mean ± SEM. Data were analyzed by one-way ANOVA, and significantly different group means (p<0.05) were then separated by the least significant difference test using GraphPad Prism (GraphPad Software, Inc.).

## Results

**Figure 1:**
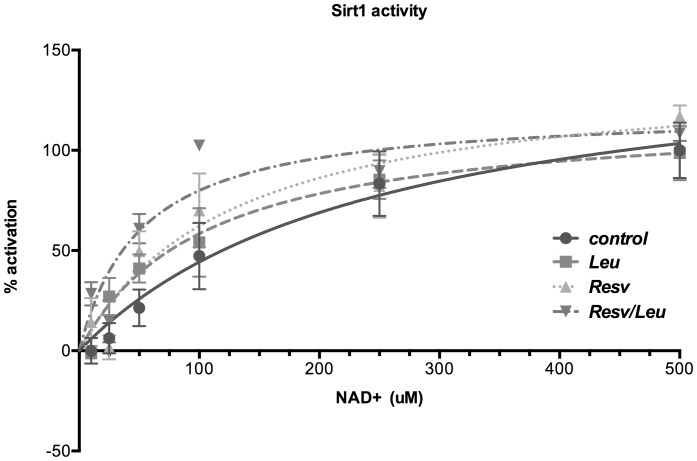
Sirt1 enzyme activity after treatment with Leucine and Resveratrol in response to changing NAD^+^ concentrations. Human recombinant Sirt1 enzyme was incubated with Leu, Resv or the combination and indicated NAD^+^ concentrations in a cell-free system using the Sirt1 FRET-based screening assay kit. Data points are represented as mean ± SEM (n = 4 to 5). Points are connected by best-fit lines using the Michaelis-Menten-Model (GraphPad Prism Software).

Figure 1 shows the activation curves of recombinant Sirt1 enzyme under different NAD^+^ concentrations when incubated with Resv, Leu or the combination Resv/Leu measured in a cell-free system using the Sirt1 FRET-based screening assay kit. Treatment with Leu significantly reduced the Km for NAD^+^ from 251 to 104 µM, while the Resv/Leu combination produced a further leftward-shift, reducing the Km to 51 µM NAD^+^ (p = 0.013).

**Figure 2:**
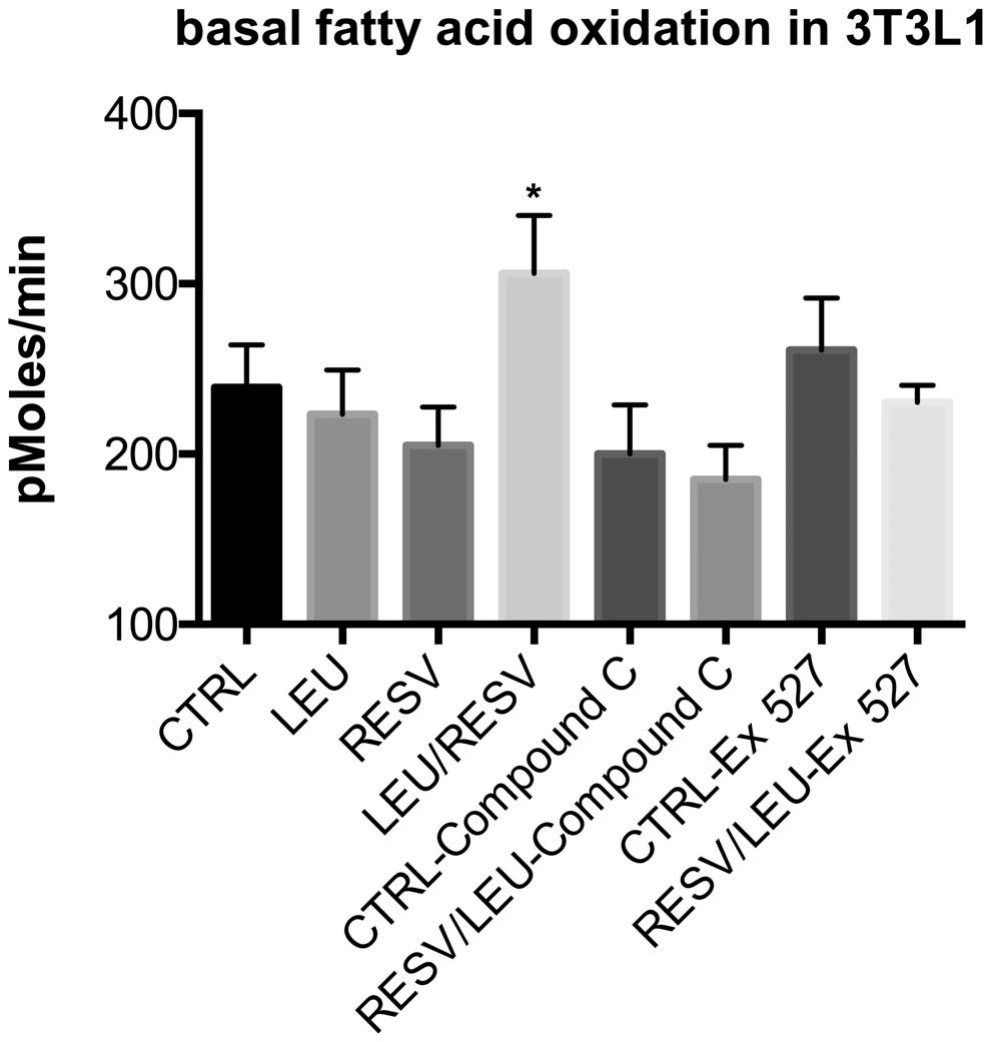
Inhibition of Leucine-Resveratrol effects on fatty acid oxidation in adipocytes by AMPK- and Sirt1 inhibitors. Differentiated 3T3L1 adipocytes were treated with indicated treatments (Leu (0.5 mM), Resv (0.2 µM), Compound C (25 µM), Ex 527 (20 µM)) for 48 h and oxygen consumption rate was measured. Basal OCR values (rate 3) were pooled together from three independent measurements for analysis. Data are represented as mean ± SEM (n = 4 to 12). Letter superscripts indicate significant difference between groups (p≤0.05).

To evaluate fatty acid oxidation, measured as the oxygen consumption rate (OCR) with the Seahorse XF analyzer, as an appropriate method of screening, we tested the effects of Resv and Leu, individually and in combination, as well as of AMPK and Sirt1 inhibitors in this system. Figure 2 shows that, consistent with our previous data, the individual concentrations of Leu and Resv had no effect on basal fatty acid oxidation in adipocytes while the combination of the two synergistically stimulated fatty acid oxidation. This effect was blocked by addition of either the AMPK inhibitor Compound C or the Sirt1 inhibitor Ex527. In addition, Resv/Leu treatment increased the palmitate-induced OCR in the model organism C. elegans (figure S1).

Subsequently, a dose-response curve for fatty acid oxidation was established for each compound studied, and a ”sub-therapeutic dose” was defined as the highest dose that exerted no effect in this system. This dose, typically found to be in the 1–100 nM range for most compounds studied, was then used to evaluate synergistic effects with Leu or HMB.

**Figure 3:**
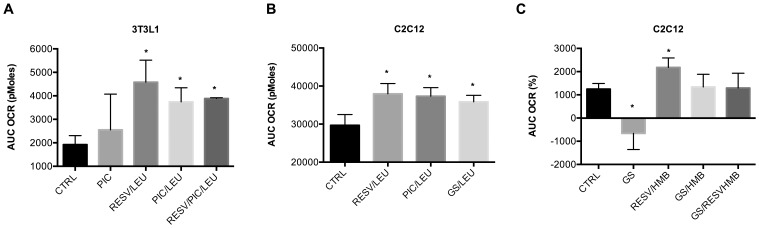
Synergistic effects of stilbenes on fatty acid oxidation in adipocytes and muscle cells. Differentiated cells were treated with indicated treatments for 24 µM palmitate injection over 4 to 5 hours. (**a**) Effects of combinations of leucine (Leu, 0.5 mM) with either resveratrol (Resv, 0.2 µM) or piceatannol (Pic, 1 nM) on oxygen consumption rate (OCR) represented as calculated areas under the curve (AUC) at a two-hour measurement point. (**b**) Comparison of the synergistic effects of Resv/Leu, grape seed extract (GS, 1 µg/ml)/Leu and Pic (1 nM)/Leu on fatty acid oxidation in C2C12 muscle cells on oxygen consumption rate (OCR) represented as calculated areas under the curve (AUC) at a two-hour measurement point. (**c**) Effects of a combination of HMB (5 µM) with Resv and GS on fatty acid oxidation in C2C12 muscle cells, represented as the AUC of OCR in % change from baseline. Data are represented as mean ± SEM (n = 4 to 5). *indicates significant difference to control (p≤0.05).

First we wanted to test if Resv related compounds such as piceatannol (Pic), a metabolite of Resv but also naturally occurring in red wine, and grape seed extract (GS), an undifferentiated mixture of polyphenols including Resv, show the same effects as Resv. As shown in figure 3, the combination of Pic with Leu had comparable effects on fat oxidation in adipocytes as the combination of Resv/Leu while no additional effect was found for the combination of Resv and Pic with Leu (figure 3b). There were also no differences between Resv/Leu, Pic/Leu or GS/Leu on fat oxidation in muscle cells (figure 3c). Similar synergistic effects were also observed for HMB with GS and Pic in adipocytes (data not shown), while no effects were detected for GS/HMB and GS/Resv/HMB (figure 3d), or for Pic/HMB and Pic/Resv/HMB (data not shown) in muscle cells.

**Figure 4:**
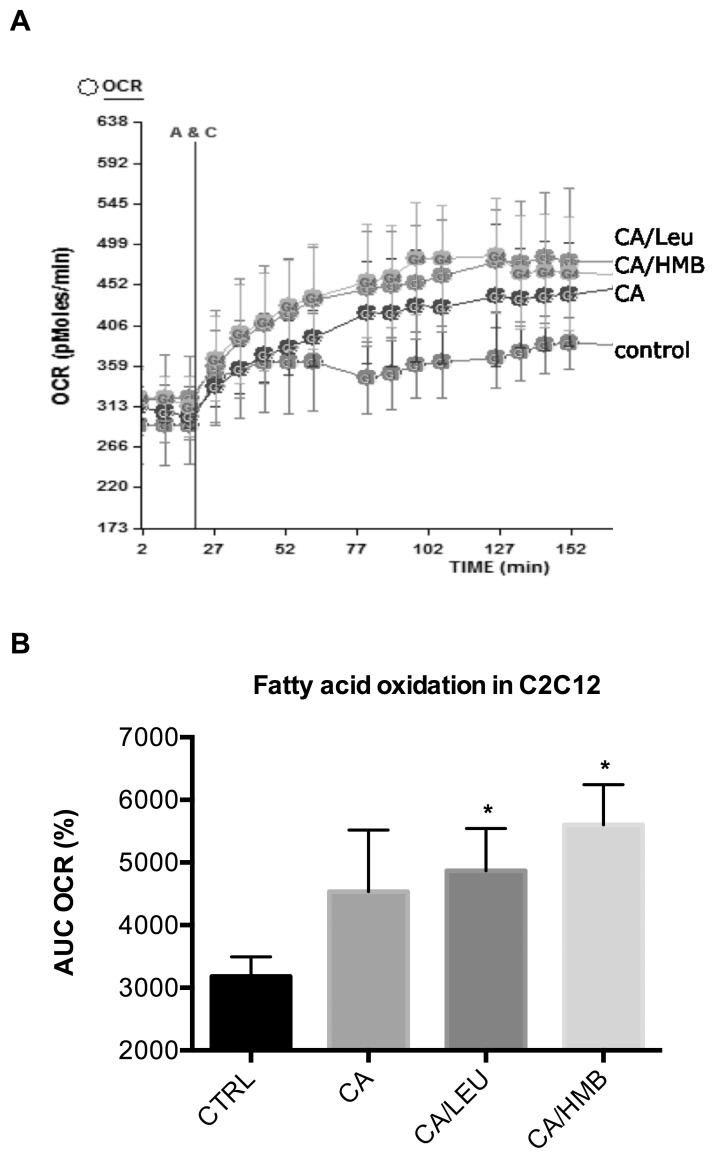
Effects of chlorogenic acid combined with either leucine or HMB on fatty acid oxidation. Differentiated cells were treated with CA (0.5 µM), Leu (0.5 µM) or HMB (5 µM) for 24 h. (**a**) Oxygen consumption rate was measured after 200 µM palmitate injection (points A&C) in C2C12 muscle cells, and (**b**) the area under the curve (AUC) at a two-hour measurement point was calculated as % change from baseline. Data are represented as mean ± SEM (n = 5). *indicates significant difference to control (p<0.05).

**Figure 5:**
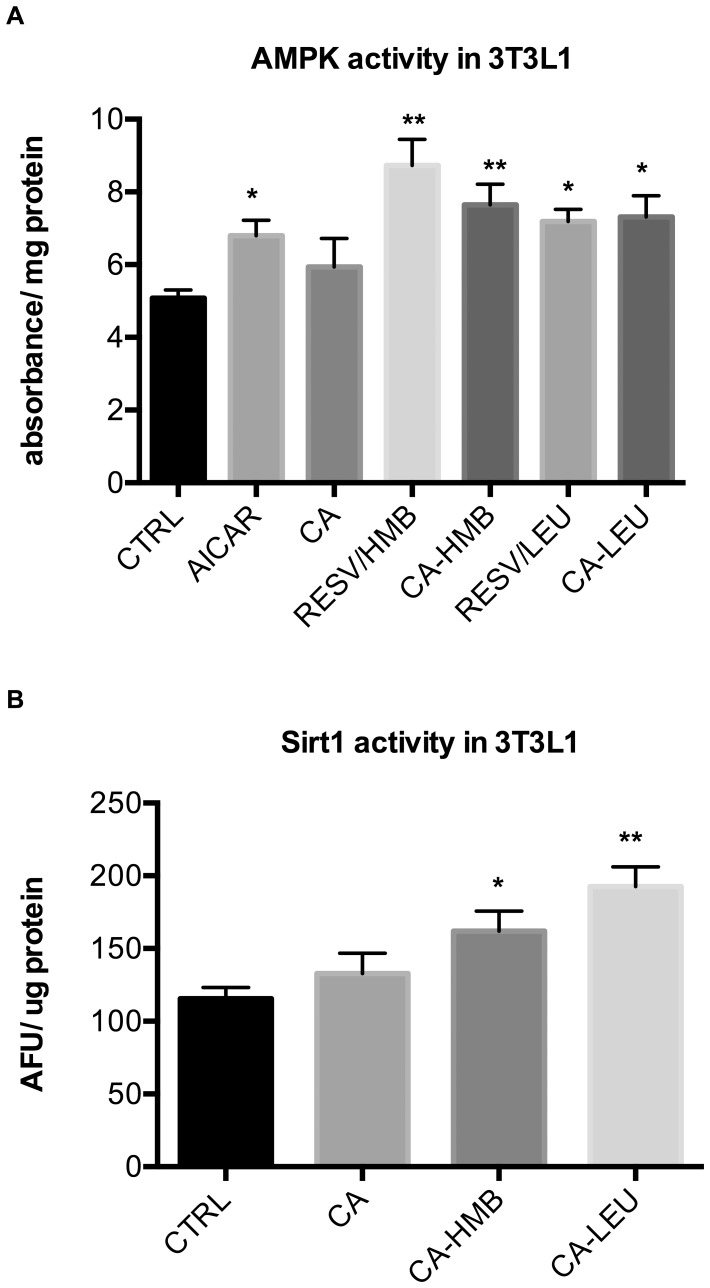
Effects of chlorogenic acid-Leu or -HMB combinations on AMPK and Sirt1 activity in adipocytes. Differentiated cells were treated with CA (0.5 µM), Leu (0.5 µM) or HMB (5 µM) (**a**) for 24 h for AMPK activity and (**b**) for 48 h for Sirt1 activity (AFU = arbitrary fluorescence units). Data are represented as mean ± SEM (n = 4). *indicates significant difference to control (p<0.03), **indicates significant difference to control and CA (p<0.05).

Next, we wanted to explore if other compounds with structure similarity to Resv exert synergy with Leu or HMB. Since Resv exists of two phenolic ring structures linked by a short carbon chain, we tested possible synergy with chlorogenic acid (CA), an ester of caffeic acid (a phenolic acid) and quinic acid (a cyclic polyol). We found that the combination CA/Leu and CA/HMB significantly increased fatty acid oxidation in muscle cells by 53 and 76%, respectively (figure 4), however, the addition of Resv to either combination attenuated these effects (data not shown). Although no effects of these combinations were observed on fatty acid oxidation in adipocytes (data not shown), treatment of adipocytes with CA/Leu and CA/HMB stimulated AMPK and Sirt1 activity up to 50% (CA/HMB) and 66% (CA/Leu), respectively (figure 5, p<0.03 for AMPK activity and p<0.001 for Sirt1 activity).

To test if two ring structures are necessary for synergistic effects, we explored next whether treatment of cells with combinations of HMB/Leu with either caffeic acid or quinic acid resulted in similar effects as seen with combinations of chlorogenic acid. While caffeic acid/Leu exerted a modest, non-statistically significant increase in muscle fatty acid oxidation, the caffeic acid-HMB combination exerted significant effects on fatty acid oxidation in both adipocytes and myotubes. Moreover, these effects were inhibited by the addition of Resv, similar to that seen with CA (figure S2). HMB or Leu combinations with quinic acid produced robust increases in adipocyte fatty acid oxidation (figure S3a). However unlike CA and caffeic acid, addition of Resv did not attenuate these effects. In myotubes, only the HMB/quinic combination increased fatty acid oxidation (figure S3b), while addition of Resv reversed this effect.

To determine if the robust effects of quinic acid reflected the effects of a unique molecule or a class of compounds, we also tested possible interactions of other cyclic and non-cyclic polyols (myo-inositol, maltitol, xylitol, sorbitol) with Leu or HMB (figure S4). However, these data did not show any significant synergistic interactions indicating that the effects of quinic acid are not readily extrapolated to other polyols.

**Figure 6:**
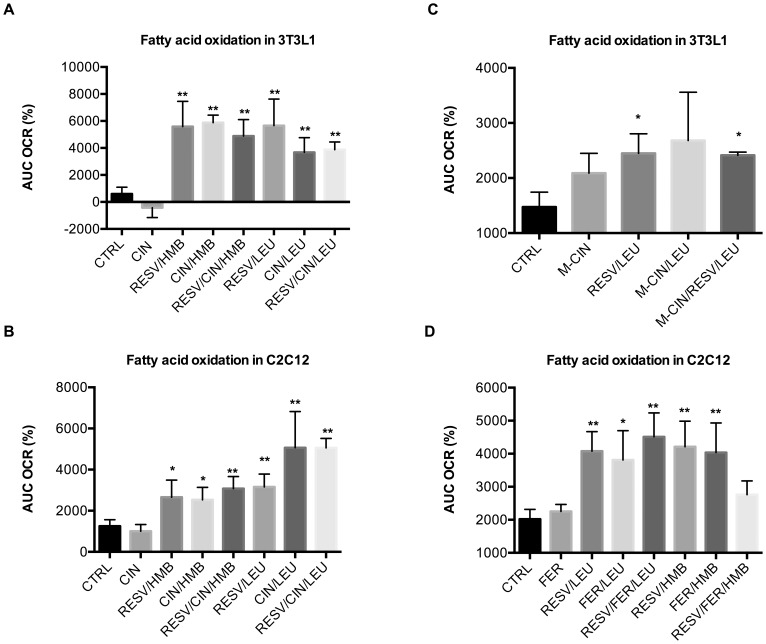
Effects of caffeic acid derivatives on fatty acid oxidation in adipocytes and muscle cells. Differentiated cells were treated with indicated treatments for 24 µM palmitate injection and the area under the curve (AUC) at a two-hour measurement point was calculated as % change from baseline. (**a**) Treatment of 3T3L1 adipocytes and (**b**) treatment of C2C12 muscle cells with combinations of Leu/HMB with cinnamic acid (Cin, 1 µM). (**c**) Treatment of 3T3L1 adipocytes with combinations of Leu with methoxy-cinnamic acid (M-Cin, 1 nM). (**d**) Treatment of C2C12 muscle cells with combinations of Leu/HMB with trans-ferulic acid (Fer, 0.5 µM). Data are represented as mean ± SEM (n = 4 to 8). *indicates significant difference to control, **indicates significant difference to control and individual compound (p<0.05).

Next we explored whether other simple phenolic acids exert synergistic effects with HMB or Leu. Since caffeic acid is a 3,4-dihydroxycinnamic acid, we started with cinnamic acid, which is a naturally occurring phenolic acid found in cinnamon oil. Cinnamic acid combined with either Leu or HMB produced robust increases in fatty acid oxidation similar to Resv/Leu and Resv/HMB both in adipocytes and muscle cells, while the addition of Resv had no further effect (figure 6a and b, p<0.05). Also combinations with trans-ferulic acid, a 4-methoxy-3-hydroxycinnamic acid, showed similar results (figure 6d), while combinations with 4-methoxycinnamic acid exerted only weaker effects (figure 6c).

**Figure 7:**
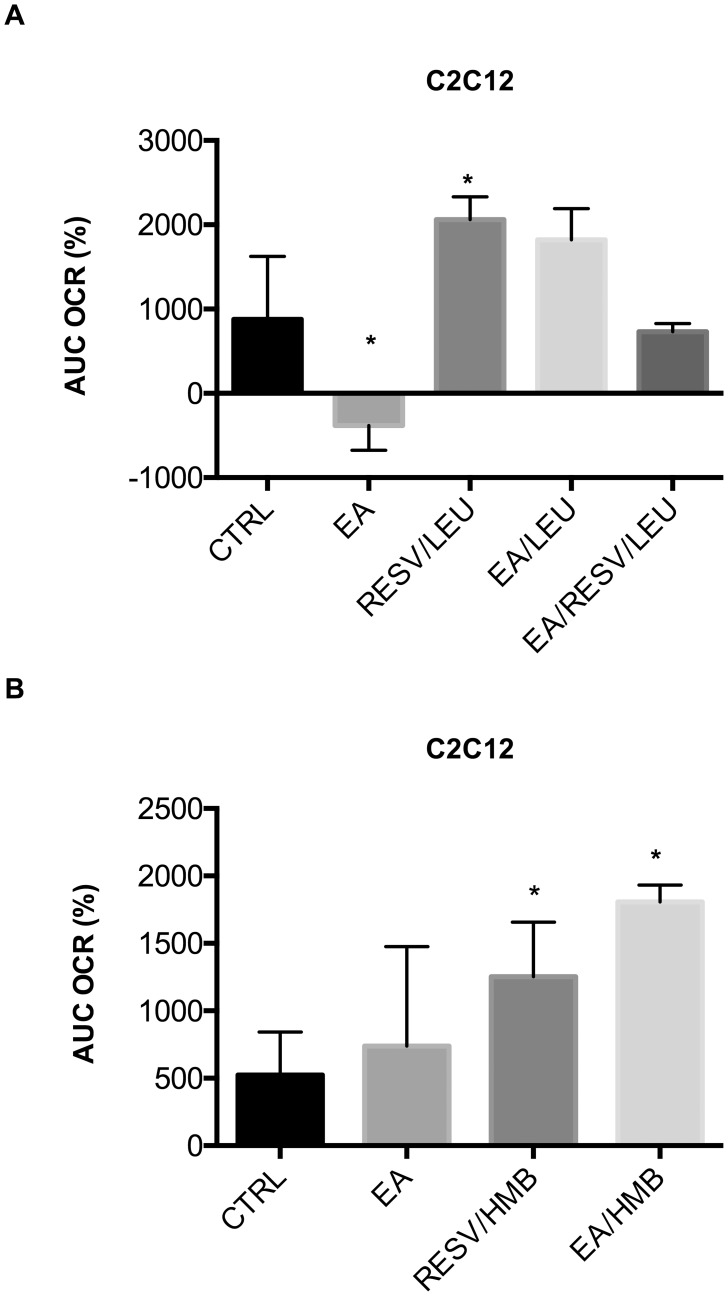
Effects of ellagic acid combinations on fatty acid oxidation in muscle cells. Differentiated C2C12 muscle cells were treated with indicated treatments for 24 µM palmitate injection and the area under the curve (AUC) at a two-hour measurement point was calculated as % change from baseline. (**a**) Ellagic acid (EA, 0.5 µM)/Leu synergy compared to Resv/Leu. (**b**) Ellagic acid (EA, 0.5 µM)/HMB Synergy compared to Resv/HMB. Data are represented as mean ± SEM (n = 4). *indicates significant difference to control (p<0.05).

We also examined possible synergy of polyphenols with a more complex structure such as epigallocatechin gallate (ECGC), the most abundant catechin in tea, and ellagic acid (EA), a tannin found in numerous fruits and vegetables. No synergistic effects were found for ECGC with Leu/HMB on fat oxidation (data not shown). EA/HMB and EA/Leu produced non-significant trends towards stimulation of fat oxidation in muscle cells (figure 7a and b; 0.05<p<0.1).

**Figure 8:**
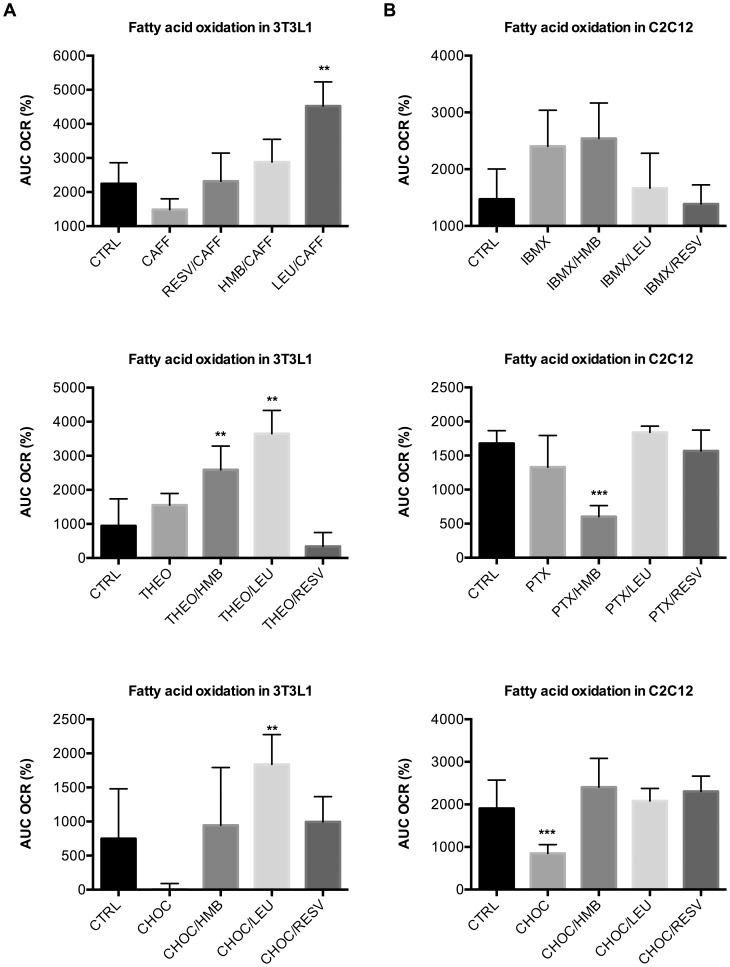
Effects of unspecific PDE inhibitors combined with Resv, Leu or HMB on fatty acid oxidation. Differentiated cells were treated with indicated treatments for 24 µM palmitate injection and the area under the curve (AUC) at a two-hour measurement point was calculated as % change from baseline. (**a**) Caffeine (Caff, 10 nM), theophylline (Theo, 1 uM) and chocamine (Choc, 0.1 ug/ml) combinations in 3T3L1 adipocytes (**b**) IBMX (50 nM), pentoxyphylline (PTX, 5 nM) and chocamine (Choc, 0.1 ug/ml) combinations in C2C12 muscle cells. Data are represented as mean ± SEM (n = 4). **indicates significant difference to control and individual compound, ***indicates significant difference to all other treatment groups (p<0.05).

Since it was recently reported that Resv’s effect on AMPK is mediated by inhibiting cAMP-phosphodiesterase [11], we tested whether Leu or HMB exert synergistic effects with other cyclic nucleotide phosphodiesterase inhibitors. First we examined non-specific PDE inhibitors; when combined with Leu, the methylxanthines caffeine and theophylline increased fatty acid oxidation in both adipocytes and muscle cells by 100 and 280%, respectively (figure 8a), but exerted no independent effects in the absence of Leu at the concentrations noted in the figure. Notably, this synergy was stronger with Leu than with HMB. Similarly, a theobromine rich cocoa extract (chocamine), exerted strong synergistic effects in combination with Leu in adipocytes (figure 8a), while no significant effects were observed in muscle cells (figure 8b). No or only weak synergistic effects were detected for IBMX and pentoxyphylline in muscle cells (figure 8b) or adipocytes (data not shown).

**Figure 9:**
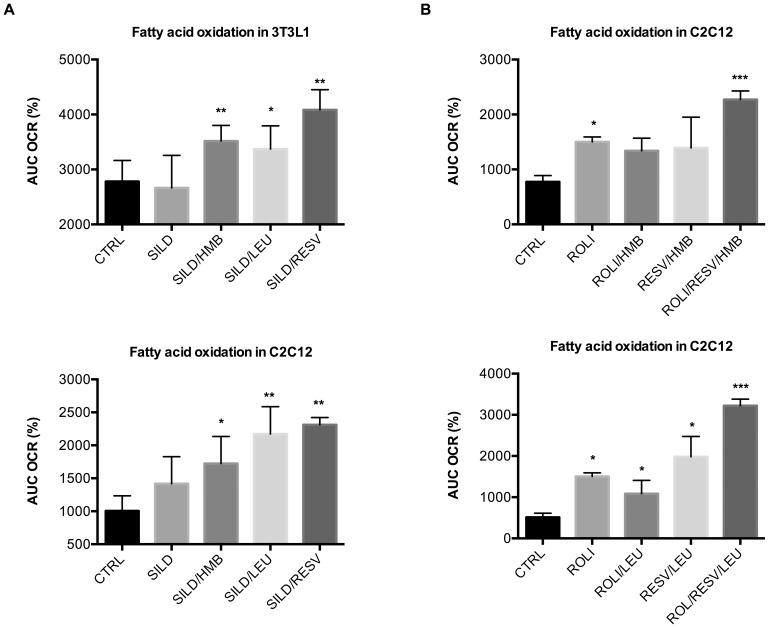
Effects of specific PDE inhibitors combined with Resv, Leu or HMB on fatty acid oxidation. Differentiated cells were treated with indicated treatments for 24 µM palmitate injection and the percent OCR from baseline (3rd measurement point) was calculated as the area under the curve (AUC) at a two-hour measurement point. (**a**) Effects of Leu/HMB/Resv combinations with the PDE5-inhibitor sildenafil (Sild, 1 nM) in 3T3L1 adipocytes and C2C12 muscle cells. (**b**) Effects of Leu/HMB/Resv combinations with the PDE4-inhibitor rolipram (Roli, 1 nM) in C2C12 muscle cells. Data are represented as mean ± SEM (n = 4 to 8). *indicates significant difference to control (p<0.05), **indicates significant difference to control and Sildenafil (p<0.05), ***indicates significant difference to all other treatment groups (p<0.05).

Next, we looked at the different classes of specific cyclic nucleotide PDE-inhibitors. While the PDE1 inhibitor vincopetine and the PDE3 inhibitors cilostamide and amrinone did not produce any significant synergistic effects with Leu/HMB on fatty acid oxidation (data not shown), we found strong significant effects for the PDE5 inhibitor sildenafil with Leu, HMB and Resv both in adipocytes and muscle cells (figure 9a). Similar synergy was also found with icariin, a naturally occurring flavinol with PDE5 inhibitory activity (figure S5). No synergistic effects were found for the c-AMP specific PDE4 inhibitors rolipram (Roli) and YM976 when combined with Leu, HMB or Resv alone. However, when Resv was added to either Roli/Leu or Roli/HMB, fatty acid oxidation was increased nearly three-fold (figure 9b).

## Discussion

These data demonstrate that Leu stimulates Sirt1 by lowering the Km for NAD^+^, enabling activation at the lower NAD^+^ concentrations characteristic of an energy replete state while potentially mimicking the response to an energy-depleted state. These effects are synergistically enhanced in the presence of Resv. Notably, the synergistic effects of both Leu and it’s metabolite, HMB, with Resv to stimulate sirtuin signaling and activation of downstream targets are not a unique feature of Resv but can also be extrapolated to other structurally related compounds, including stilbenes and hydroxycinnamic acids. These effects occur at concentrations that produce no independent effects and can be readily achieved via diet or supplementation. Moreover, some of these synergies were more robust than those we previously found for low-dose Resv and Leu.

A number of polyphenols exert salutary effects on glucose and lipid metabolism, suggesting utility in the prevention and treatment of diabetes [17]–[23]. However, the concentrations used in these studies are generally manifold higher than those found in human plasma following oral dosing with either diet or supplements. Since most compounds have a low bioavailability and are rapidly metabolized by glucuronidation, methylation and sulfation, thus increasing their excretion and further reducing their total plasma concentration [24], [25], an impractically high intake would be required to achieve those µM concentrations used in these studies. Indeed, a lower dose of Resv was recently found to exert no significant effect on metabolic function in non-obese women [21]. However, the observation that Leu acts synergistically with Resv on metabolic outcomes such as insulin sensitivity and fat oxidation [14], reduces the necessary concentration to low levels that can easily be achieved by diet. This synergistic action is most likely caused by allosteric activation of Sirt1 by Leu. Although the direct effects of Resv on Sirt1 activation has been linked to the fluorophore used in the activity assay [26], recent evidence demonstrates that, depending on the substrate-Resv fit, the fluorophore in the assay is necessary to connect the substrate with Resv [27] and that endogenously present hydrophobic amino acids may substitute for the fluorophore [10]. Therefore, the highly hydrophobic amino acid Leu may replace the fluorophore and act as an allosteric activator of Sirt1. Consistent with this, we found Leu activation of Sirt1 and Leu-Resv synergy in a FRET-based assay free of fluorophore binding artifact.

We found the ability of Leu to amplify Resv stimulation of Sirt1 to be extrapolated to other polyphenols with structural similarity. Chlorogenic acid, a phenolic acid consisting of an ester of hydroxycinnamic acid and quinic acid, exerted no significant independent effects on fat oxidation in muscle cells and AMPK and Sirt1 activity in adipocytes at a concentration of 500 nmol/L, which is achieved in plasma after one cup of coffee containing 96 mg of chlorogenic acid [28]. However, this concentration exhibited significant synergy with Leu and HMB in stimulating these responses. Interestingly, the addition of Resv to these combinations attenuated these effects, suggesting potential competition with chlorogenic acid for a common site of action. We found also similar robust synergistic action with other hydroxycinnamic acids, including cinnamic acid and its derivatives ferulic acid and p-methoxy-cinnamic acid, as well with quinic acid but not with other polyols suggesting that the six-carbon ring structure bound to a carboxylic group plays an important role in the action of polyphenols in this system.

Since recent data [11] indicate that, rather than having a direct effect on Sirt1, Resv-induced activation may be mediated, in part, via inhibiting cAMP phosphodiesterase, resulting in upregulation of AMPK and subsequent activation of Sirt1, we evaluated the effects of naturally occurring as well as of specific pharmaceutical PDE inhibitors in combination with Leu or HMB on fat oxidation and AMPK/Sirt activation. We also tested both non-specific and specific PDE inhibitors. Our data indicate strong synergistic effects between Leu and the non-specific methylxanthines caffeine, theophylline and theobromine. Theophylline has been used for the treatment of asthma since 1937; however because of its narrow therapeutic range, the use of theophylline has decreased over the years with the development of newer more selective drugs. The bronchodilatory actions of theophylline require therapeutic plasma concentrations between 27 and 55 uM [29], [30]. To address the potential for lower concentrations of theophylline to exert therapeutic effects in combination with Leu, we assessed the impact of these treatments on inflammatory markers in mouse primary lung endothelial cells (Cell Biologics, Chicago, IL). Although 1 uM theophylline exerted no independent effect on nuclear factor –kappa B (NF-κB) protein expression, theophylline/Leu and theophylline/Leu/Resv combinations resulted in significant decreases (p<0.02, figure S6a). Moreover, Leu/theophylline combinations reduced interleukin 1- beta (IL1- β) release (∼50%, p<0.025) from mouse lung endothelial cells (figure S6b). These data suggest that synergy with Leu may be a useful strategy to expand the therapeutic range of theophylline to lower concentrations and thereby minimize likelihood of adverse events.

Some of the action of the methylxanthines is also mediated by adenosine receptor antagonism; accordingly we evaluated the synergistic effects of specific PDE inhibitors on metabolic outcomes. Effects of PDE inhibitors on body weight and fat oxidation has been reported previously. Particularly PDE3 and PDE4 inhibitors are believed to play a role in cAMP regulated adipocyte lipolysis since they provide the major cAMP hydrolyzing activity in most tissues [31]. Theophylline and the PDE 3 inhibitor amrinone was shown to increase adipocyte lipolysis dose-dependently to a maximum of 200% and 63%, respectively, measured by microdialysis technique of human abdominal adipose tissue [32]. In addition, the combined effect was not different from that with theophylline alone suggesting that the selective action of amrinone is part of the non-selective action of theophylline [32]. PDE4B deficient mice showed reduced adiposity with lower white fat pads weights and smaller adipocytes both on chow and high-fat-diet [33]. Although we found an increased fat oxidation for most tested PDE inhibitors individually, synergistic actions with Leu or HMB was detected only for the non-specific PDE inhibitors and PDE 5 inhibitors.

PDE5 is selective for cGMP, and its inhibition does not lead to AMPK activation. However, PDE5 inhibitors stimulate nitric oxide (NO) signaling and are therefore effective vasodilators. As such, PDE5 inhibitors such as Sildenafil are mainly used for treatment of erectile dysfunction and pulmonary hypertension. However, evidence shows that stimulation of NO-c-GMP signaling also modulates energy metabolism in mouse skeletal muscle *in vitro* and i*n vivo* [34], [35], and is associated with increased PGC-1α stimulated mitochondrial biogenesis [36]–[39]. Moreover Mischke et al. [40] reported recently, that treatment of primary adipocytes with cGMP resulted in increased adipogenesis and the development of a brown-like thermogenic program with upregulation of uncoupling protein 1 (UCP1) expression. These effects were also observed *in vivo* after treatment of C57BL/6 mice with sildenafil for seven days. We found significant increases of NO production and mitochondrial mass for both PDE5 inhibitors, sildenafil and icariin, in combination with Leu and HMB (figure S7), suggesting that the synergistic actions of these compounds may converge on this pathway.

## Conclusion

These data demonstrate that the synergistic action between Leu and Resv is not unique but also can be extrapolated to other polyphenols with structural similarities. The single aromatic ring structure bound to a carboxylic group appears to play a key structural role for this synergy. In addition, Leu signaling may also synergize with compounds such as methylxanthines and PDE5 inhibitors on other AMPK-independent pathways, such as NO signaling to modulate energy metabolism.

## Supporting Information

Figure S1 Synergistic effects of RESV/LEU on fatty acid oxidation in *C. elegans.* Synchronized L1 worms were maintained in liquid media. When they reached L3 stage, they were treated with Resv (0.2 µM)/Leu (0.5 mM) or vehicle for 48 h. Oxygen consumption rate (OCR) was measured after 200 µM palmitate injection. Data are represented as mean ± SEM (n = 10) of calculated areas under the curve (AUC) of OCR in % change from baseline at a two-hour measurement point. *indicates significant difference to control.
(TIFF)

Figure S2 Synergistic effects of Caffeic acid on fatty acid oxidation in 3T3L1 adipocytes and C2C12 muscle cells. Differentiated cells were treated with indicated treatments for 24 h. Oxygen consumption rate (OCR) was measured after 200 µM palmitate injection. Effects of combinations of HMB or Leu with caffeic acid (CAFF, 1 µM) on OCR in **(a)** C2C12 muscle cells and **(b)** 3T3L1 adipocytes. Data are represented as mean ± SEM (n = 4) of calculated areas under the curve (AUC) of OCR in % change from baseline at a two-hour measurement point. *indicates significant difference to control, **indicates significant difference to control and CAFF (p≤0.05).
(TIFF)

Figure S3 Synergistic effects of Quinic acid on fatty acid oxidation in 3T3L1 adipocytes and C2C12 muscle cells. Differentiated cells were treated with indicated treatments for 24 h. Oxygen consumption rate (OCR) was measured after 200 µM palmitate injection. Effects of combinations of HMB or Leu with quinic acid (QA, 0.5 µM) on OCR in **(a)** 3T3L1 adipocytes and **(b)** C2C12 muscle cells. Data are represented as mean ± SEM (n = 4) of calculated areas under the curve (AUC) of OCR in % change from baseline at a two-hour measurement point. *indicates significant difference to control, **indicates significant difference to control and QA (p≤0.05).
(TIFF)

Figure S4 Effects of Leu, HMB or Resv combined with polyols on fatty acid oxidation in C2C12 muscle cells. Differentiated cells were treated with indicated treatments for 24 h. Oxygen consumption rate (OCR) was measured after 200 µM palmitate injection. Effects of combinations of Leu, HMB or Resv with **(a)** Maltitol (MALT, 0.1 µM), **(b)** Sorbitol (SORB, 0.5 µM), **(c)** Xylitol (Xyl, 10 nM) and **(d)** myo-Inositol (MYO, 0.1 µM) on OCR C2C12 muscle cells. Data are represented as mean ± SEM (n = 4) of calculated areas under the curve (AUC) of OCR in % change from baseline at a two-hour measurement point. *indicates significant difference to control (p≤0.05).
(TIFF)

Figure S5 Synergistic effects of Icariin on fatty acid oxidation in 3T3L1 adipocytes and C2C12 muscle cells. Differentiated cells were treated with indicated treatments for 24 h. Oxygen consumption rate (OCR) was measured after 200 µM palmitate injection. Effects of combinations of Leu, HMB or Resv with Icariin (Icar, 1 nM) on OCR in **(a)** 3T3L1 adipocytes and **(b)** C2C12 muscle cells. Data are represented as mean ± SEM (n = 4) of calculated areas under the curve (AUC) of OCR in pMoles at a two-hour measurement point. *indicates significant difference to control, **indicates significant difference to control and icariin (p≤0.05).
(TIFF)

Figure S6 Effects of theophylline-Resv-Leu-combinations on inflammatory biomarker in mouse lung endothelial cells. Mouse lung endothelial cells were treated with 1 µM theophylline alone or in combination with leucine and resveratrol for 24 h. TNF-α (10 ng/ml) was used as positive control. **(a)** Phospho-NF-κB-expression was determined by Western blot in cell lysate using anti-Phospho-NF-κB antibody (Cell Signaling, Billerica, MA, USA) and bands were quantified using Image Lab Software (Bio-Rad, Hercules, CA, USA). **(b)** IL-1β release in cell culture media determined via IL-1β ELISA kit (Abcam, Cambridge, MA, USA). Data are represented as mean ± SEM (n = 2 to 4). *indicates significant difference to control (p<0.05).
(TIFF)

Figure S7 Synergistic effects of Sildenafil and Icariin on nitric oxide (NO) production in C2C12 muscle cells. Differentiated cells were treated with indicated treatments for 4 h. NO production was detected by fluorescence using the fluorophore diaminofluorescein diacetate DAF-2DA (Cell Technology, Inc.). **(a)** Effects of Sildenafil (Sild, 1 nM) and **(b)** of Icariin (Icar, 1 nM) combinations with Leu, HMB or Resv in C2C12 muscle cells. Data are represented as mean ±SEM (n = 6). *indicates significant difference to control (p≤0.05), **indicates significant difference to control and Sild (a), or control and Icariin (b) (p≤0.05).
(TIFF)

## References

[1] Milne JC, Lambert PD, Schenk S, Carney DP, Smith JJ, et al (2007) Small molecule activators of SIRT1 as therapeutics for the treatment of type 2 diabetes. Nature 450: 712–716. 18046409 10.1038/nature06261 PMC2753457

[2] Yoshizaki T, Milne JC, Imamura T, Schenk S, Sonoda N, et al (2009) SIRT1 exerts anti-inflammatory effects and improves insulin sensitivity in adipocytes. Mol Cell Biol 29: 1363–1374. 19103747 10.1128/MCB.00705-08 PMC2643824

[3] Canto C, Auwerx J (2009) PGC-1alpha, SIRT1 and AMPK, an energy sensing network that controls energy expenditure. Curr Opin Lipidol 20: 98–105. 19276888 10.1097/MOL.0b013e328328d0a4 PMC3627054

[4] Jager S, Handschin C, St-Pierre J, Spiegelman BM (2007) AMP-activated protein kinase (AMPK) action in skeletal muscle via direct phosphorylation of PGC-1alpha. Proc Natl Acad Sci U S A 104: 12017–12022. 17609368 10.1073/pnas.0705070104 PMC1924552

[5] Brenmoehl J, Hoeflich A (2013) Dual control of mitochondrial biogenesis by sirtuin 1 and sirtuin 3. Mitochondrion. 10.1016/j.mito.2013.04.002 23583953

[6] Timmer S, Auwerx J, Schrauwen P (2012) The journey of resveratrol from yeast to human. Aging (Albany NY) 4: 146–158. 22436213 10.18632/aging.100445 PMC3348475

[7] Pearson KJ, Baur JA, Lewis KN, Peshkin L, Price NL, et al (2008) Resveratrol delays age-related deterioration and mimics transcriptional aspects of dietary restriction without extending life span. Cell Metab 8: 157–168. 18599363 10.1016/j.cmet.2008.06.011 PMC2538685

[8] Borra MT, Smith BC, Denu JM (2005) Mechanism of human SIRT1 activation by resveratrol. J Biol Chem 280: 17187–17195. 15749705 10.1074/jbc.M501250200

[9] Pacholec M, Bleasdale JE, Chrunyk B, Cunningham D, Flynn D, et al (2010) SRT1720, SRT2183, SRT1460, and resveratrol are not direct activators of SIRT1. J Biol Chem 285: 8340–8351. 20061378 10.1074/jbc.M109.088682 PMC2832984

[10] Hubbard BP, Gomes AP, Dai H, Li J, Case AW, et al (2013) Evidence for a common mechanism of SIRT1 regulation by allosteric activators. Science 339: 1216–1219. 23471411 10.1126/science.1231097 PMC3799917

[11] Park SJ, Ahmad F, Philp A, Baar K, Williams T, et al (2012) Resveratrol ameliorates aging-related metabolic phenotypes by inhibiting cAMP phosphodiesterases. Cell 148: 421–433. 22304913 10.1016/j.cell.2012.01.017 PMC3431801

[12] Price NL, Gomes AP, Ling AJ, Duarte FV, Martin-Montalvo A, et al (2012) SIRT1 Is Required for AMPK Activation and the Beneficial Effects of Resveratrol on Mitochondrial Function. Cell Metab 15: 675–690. 22560220 10.1016/j.cmet.2012.04.003 PMC3545644

[13] Bruckbauer A, Zemel MB (2011) Effects of dairy consumption on SIRT1 and mitochondrial biogenesis in adipocytes and muscle cells. Nutr Metab (Lond) 8: 91. 22185590 10.1186/1743-7075-8-91 PMC3264668

[14] Bruckbauer A, Zemel MB, Thorpe T, Akula MR, Stuckey AC, et al (2012) Synergistic effects of leucine and resveratrol on insulin sensitivity and fat metabolism in adipocytes and mice. Nutr Metab (Lond) 9: 77. 22913271 10.1186/1743-7075-9-77 PMC3506499

[15] Feige JN, Lagouge M, Canto C, Strehle A, Houten SM, et al (2008) Specific SIRT1 activation mimics low energy levels and protects against diet-induced metabolic disorders by enhancing fat oxidation. Cell Metab 8: 347–358. 19046567 10.1016/j.cmet.2008.08.017

[16] Nin V, Escande C, Chini CC, Giri S, Camacho-Pereira J, et al (2012) Role of Deleted in Breast Cancer 1 (DBC1) Protein in SIRT1 Deacetylase Activation Induced by Protein Kinase A and AMP-activated Protein Kinase. J Biol Chem 287: 23489–23501. 22553202 10.1074/jbc.M112.365874 PMC3390625

[17] Ong KW, Hsu A, Tan BK (2012) Chlorogenic acid stimulates glucose transport in skeletal muscle via AMPK activation: a contributor to the beneficial effects of coffee on diabetes. PLoS One 7: e32718. 22412912 10.1371/journal.pone.0032718 PMC3296733

[18] Cho AS, Jeon SM, Kim MJ, Yeo J, Seo KI, et al (2010) Chlorogenic acid exhibits anti-obesity property and improves lipid metabolism in high-fat diet-induced-obese mice. Food Chem Toxicol 48: 937–943. 20064576 10.1016/j.fct.2010.01.003

[19] Huang B, Yuan HD, Kim do Y, Quan HY, Chung SH (2011) Cinnamaldehyde prevents adipocyte differentiation and adipogenesis via regulation of peroxisome proliferator-activated receptor-gamma (PPARgamma) and AMP-activated protein kinase (AMPK) pathways. J Agric Food Chem 59: 3666–3673. 21401097 10.1021/jf104814t

[20] Kang W, Hong HJ, Guan J, Kim DG, Yang EJ, et al (2012) Resveratrol improves insulin signaling in a tissue-specific manner under insulin-resistant conditions only: in vitro and in vivo experiments in rodents. Metabolism 61: 424–433. 21945106 10.1016/j.metabol.2011.08.003

[21] Robich MP, Osipov RM, Chu LM, Han Y, Feng J, et al (2011) Resveratrol modifies risk factors for coronary artery disease in swine with metabolic syndrome and myocardial ischemia. Eur J Pharmacol 664: 45–53. 21575630 10.1016/j.ejphar.2011.04.059 PMC3107708

[22] Brasnyo P, Molnar GA, Mohas M, Marko L, Laczy B, et al (2011) Resveratrol improves insulin sensitivity, reduces oxidative stress and activates the Akt pathway in type 2 diabetic patients. Br J Nutr 106: 383–389. 21385509 10.1017/S0007114511000316

[23] Cho SJ, Jung UJ, Choi MS (2012) Differential effects of low-dose resveratrol on adiposity and hepatic steatosis in diet-induced obese mice. Br J Nutr: 1–10. 10.1017/S0007114512000347 22414733

[24] D'Archivio M, Filesi C, Vari R, Scazzocchio B, Masella R (2010) Bioavailability of the polyphenols: status and controversies. Int J Mol Sci 11: 1321–1342. 20480022 10.3390/ijms11041321 PMC2871118

[25] Boocock DJ, Faust GE, Patel KR, Schinas AM, Brown VA, et al (2007) Phase I dose escalation pharmacokinetic study in healthy volunteers of resveratrol, a potential cancer chemopreventive agent. Cancer Epidemiol Biomarkers Prev 16: 1246–1252. 17548692 10.1158/1055-9965.EPI-07-0022

[26] Kaeberlein M, McDonagh T, Heltweg B, Hixon J, Westman EA, et al (2005) Substrate-specific activation of sirtuins by resveratrol. J Biol Chem 280: 17038–17045. 15684413 10.1074/jbc.M500655200

[27] Gertz M, Nguyen GT, Fischer F, Suenkel B, Schlicker C, et al (2012) A molecular mechanism for direct sirtuin activation by resveratrol. PLoS One 7: e49761. 23185430 10.1371/journal.pone.0049761 PMC3504108

[28] Manach C, Scalbert A, Morand C, Remesy C, Jimenez L (2004) Polyphenols: food sources and bioavailability. Am J Clin Nutr 79: 727–747. 15113710 10.1093/ajcn/79.5.727

[29] Boswell-Smith V, Cazzola M, Page CP (2006) Are phosphodiesterase 4 inhibitors just more theophylline? J Allergy Clin Immunol 117: 1237–1243. 16750981 10.1016/j.jaci.2006.02.045

[30] Baraldi PG, Tabrizi MA, Gessi S, Borea PA (2008) Adenosine receptor antagonists: translating medicinal chemistry and pharmacology into clinical utility. Chem Rev 108: 238–263. 18181659 10.1021/cr0682195

[31] Francis SH, Blount MA, Corbin JD (2011) Mammalian cyclic nucleotide phosphodiesterases: molecular mechanisms and physiological functions. Physiol Rev 91: 651–690. 21527734 10.1152/physrev.00030.2010

[32] Arner P, Hellmer J, Hagstrom-Toft E, Bolinder J (1993) Effect of phosphodiesterase inhibition with amrinone or theophylline on lipolysis and blood flow in human adipose tissue in vivo as measured with microdialysis. J Lipid Res 34: 1737–1743. 8245724

[33] Zhang R, Maratos-Flier E, Flier JS (2009) Reduced adiposity and high-fat diet-induced adipose inflammation in mice deficient for phosphodiesterase 4B. Endocrinology 150: 3076–3082. 19359377 10.1210/en.2009-0108 PMC2703511

[34] Sabatini S, Sgro P, Duranti G, Ceci R, Di Luigi L (2011) Tadalafil alters energy metabolism in C2C12 skeletal muscle cells. Acta Biochim Pol 58: 237–241. 21681286

[35] Ayala JE, Bracy DP, Julien BM, Rottman JN, Fueger PT, et al (2007) Chronic treatment with sildenafil improves energy balance and insulin action in high fat-fed conscious mice. Diabetes 56: 1025–1033. 17229936 10.2337/db06-0883

[36] Nisoli E, Clementi E, Paolucci C, Cozzi V, Tonello C, et al (2003) Mitochondrial biogenesis in mammals: the role of endogenous nitric oxide. Science 299: 896–899. 12574632 10.1126/science.1079368

[37] Nisoli E, Falcone S, Tonello C, Cozzi V, Palomba L, et al (2004) Mitochondrial biogenesis by NO yields functionally active mitochondria in mammals. Proc Natl Acad Sci U S A 101: 16507–16512. 15545607 10.1073/pnas.0405432101 PMC534517

[38] De Toni L, Strapazzon G, Gianesello L, Caretta N, Pilon C, et al (2011) Effects of type 5-phosphodiesterase inhibition on energy metabolism and mitochondrial biogenesis in human adipose tissue ex vivo. J Endocrinol Invest 34: 738–741. 22234177 10.1007/BF03346724

[39] Lira VA, Brown DL, Lira AK, Kavazis AN, Soltow QA, et al (2010) Nitric oxide and AMPK cooperatively regulate PGC-1 in skeletal muscle cells. J Physiol 588: 3551–3566. 20643772 10.1113/jphysiol.2010.194035 PMC2988518

[40] Mitschke MM, Hoffmann LS, Gnad T, Scholz D, Kruithoff K, et al (2013) Increased cGMP promotes healthy expansion and browning of white adipose tissue. FASEB J 27: 1621–1630. 23303211 10.1096/fj.12-221580

