# Supplementary figures and images for: Synergistic Effects of Polyphenols and Methylxanthines with Leucine on AMPK/Sirtuin-Mediated Metabolism in Muscle Cells and Adipocytes
Source: PLoS One. 2014 Feb 14;9(2):e89166. doi: 10.1371/journal.pone.0089166 (PMC3925247; doi:10.1371/journal.pone.0089166)

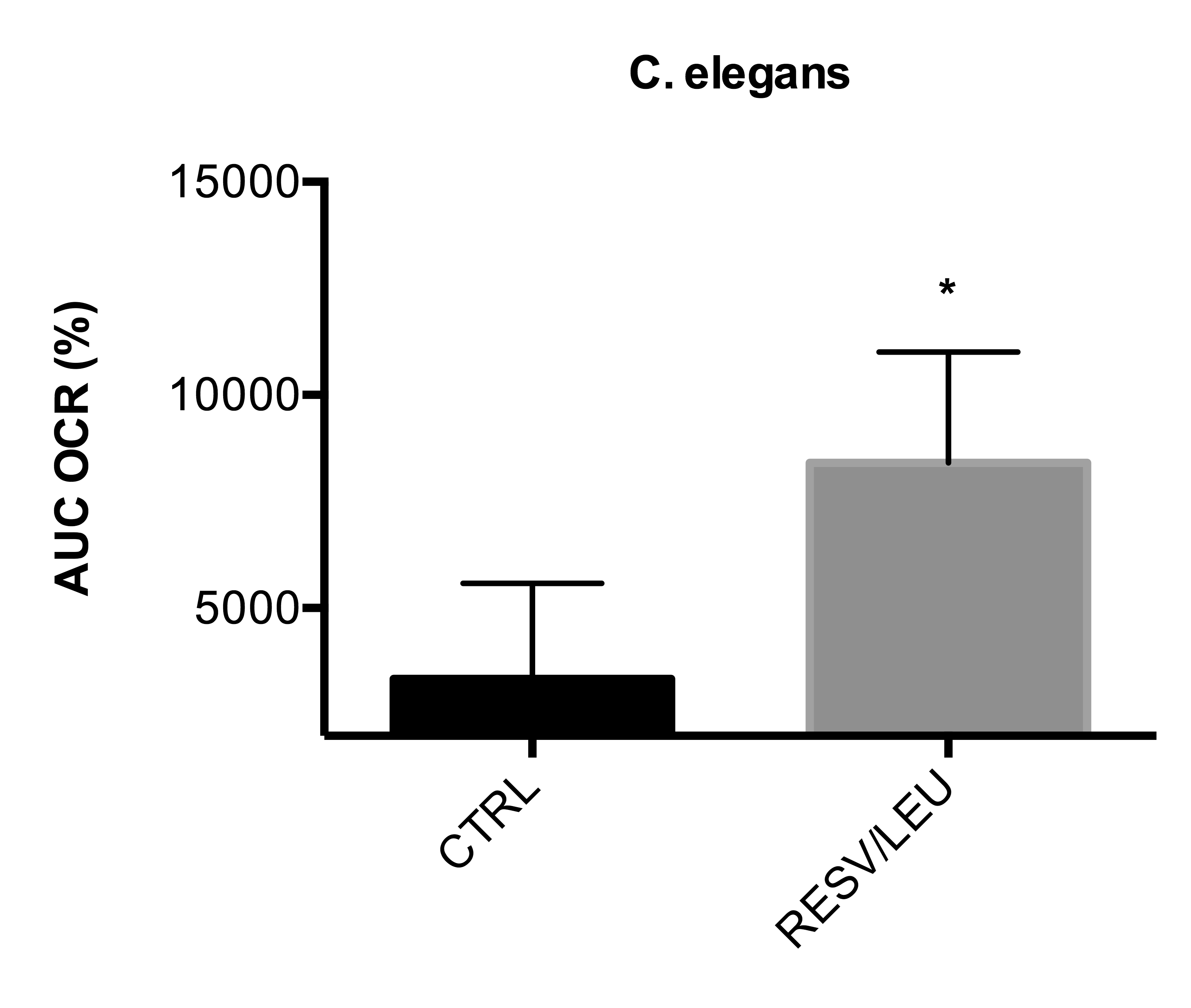

Supplement: Figure S1 — Synergistic effects of RESV/LEU on fatty acid oxidation in C. elegans. Synchronized L1 worms were maintained in liquid media. When they reached L3 stage, they were treated with Resv (0.2 µM)/Leu (0.5 mM) or vehicle for 48 h. Oxygen consumption rate (OCR) was measured after 200 µM palmitate injection. Data are represented as mean ± SEM (n = 10) of calculated areas under the curve (AUC) of OCR in % change from baseline at a two-hour measurement point. *indicates significant difference to control. (TIFF) [file pone.0089166.s001.tif]

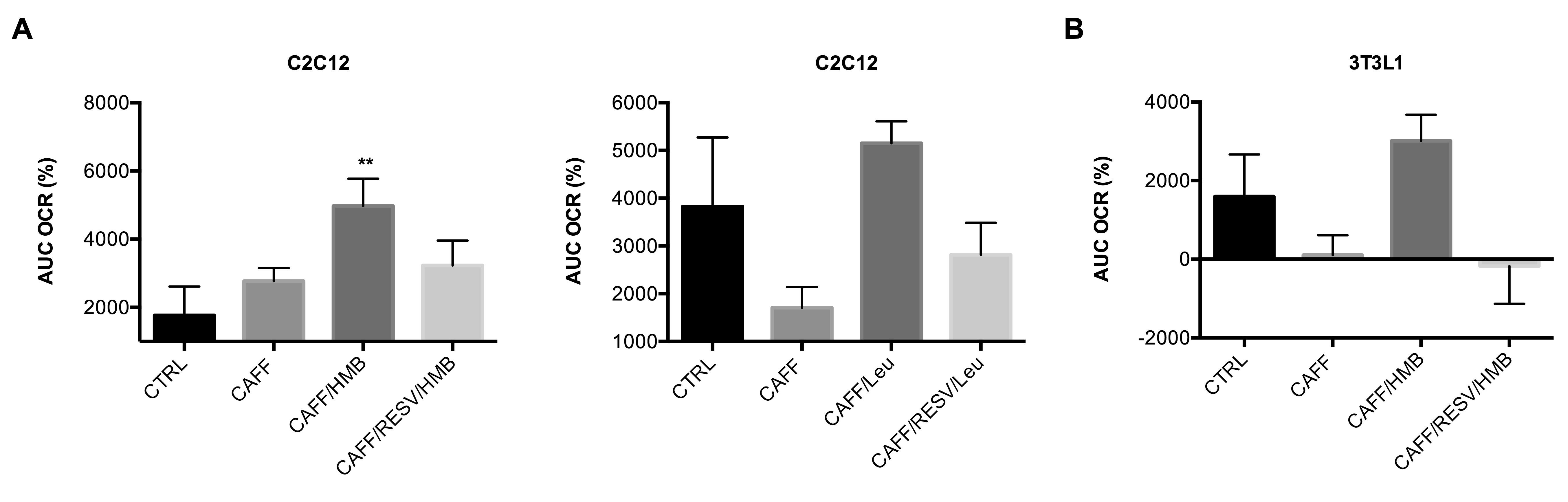

Supplement: Figure S2 — Synergistic effects of Caffeic acid on fatty acid oxidation in 3T3L1 adipocytes and C2C12 muscle cells. Differentiated cells were treated with indicated treatments for 24 h. Oxygen consumption rate (OCR) was measured after 200 µM palmitate injection. Effects of combinations of HMB or Leu with caffeic acid (CAFF, 1 µM) on OCR in (a) C2C12 muscle cells and (b) 3T3L1 adipocytes. Data are represented as mean ± SEM (n = 4) of calculated areas under the curve (AUC) of OCR in % change from baseline at a two-hour measurement point. *indicates significant difference to control, **indicates significant difference to control and CAFF (p≤0.05). (TIFF) [file pone.0089166.s002.tif]

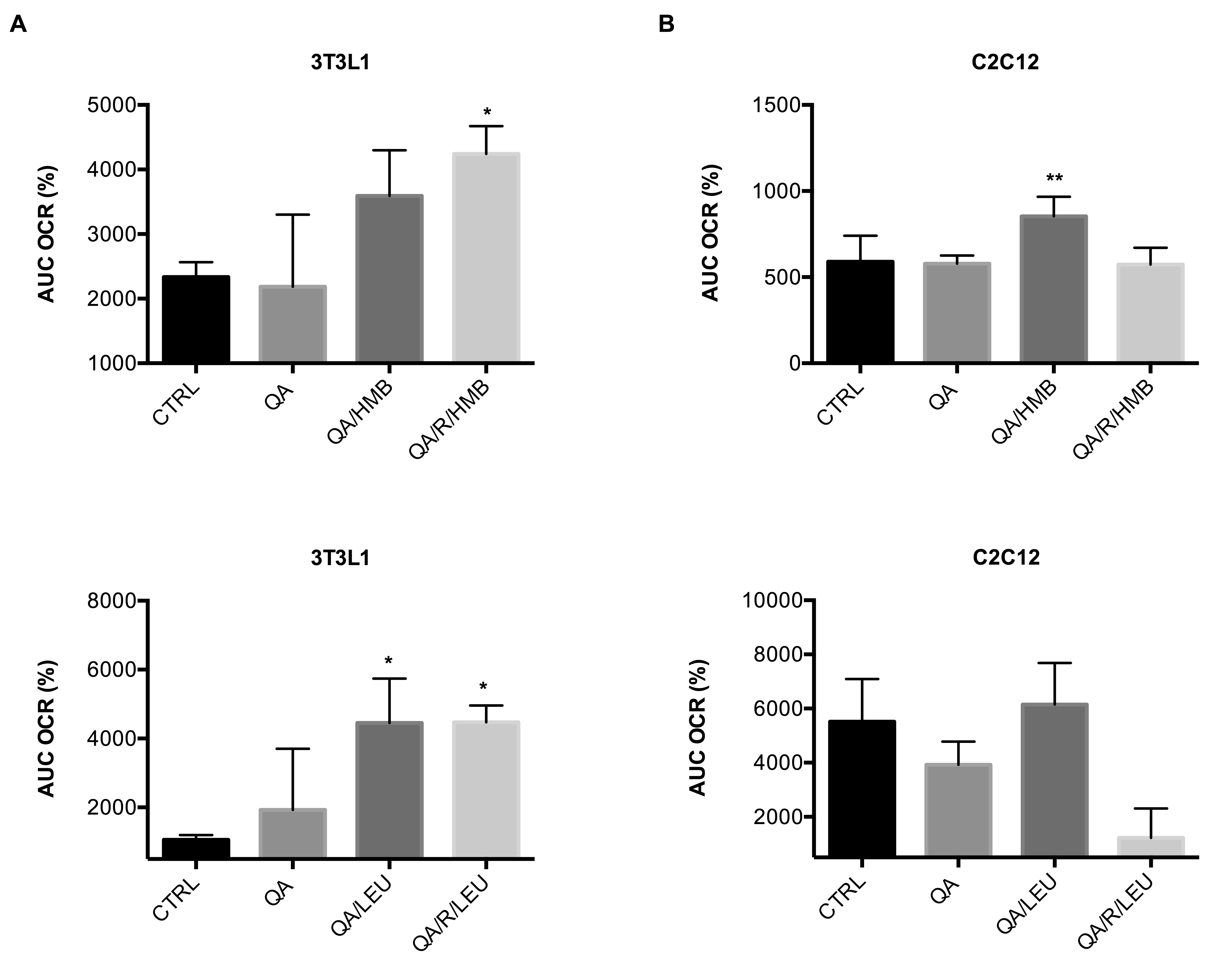

Supplement: Figure S3 — Synergistic effects of Quinic acid on fatty acid oxidation in 3T3L1 adipocytes and C2C12 muscle cells. Differentiated cells were treated with indicated treatments for 24 h. Oxygen consumption rate (OCR) was measured after 200 µM palmitate injection. Effects of combinations of HMB or Leu with quinic acid (QA, 0.5 µM) on OCR in (a) 3T3L1 adipocytes and (b) C2C12 muscle cells. Data are represented as mean ± SEM (n = 4) of calculated areas under the curve (AUC) of OCR in % change from baseline at a two-hour measurement point. *indicates significant difference to control, **indicates significant difference to control and QA (p≤0.05). (TIFF) [file pone.0089166.s003.tif]

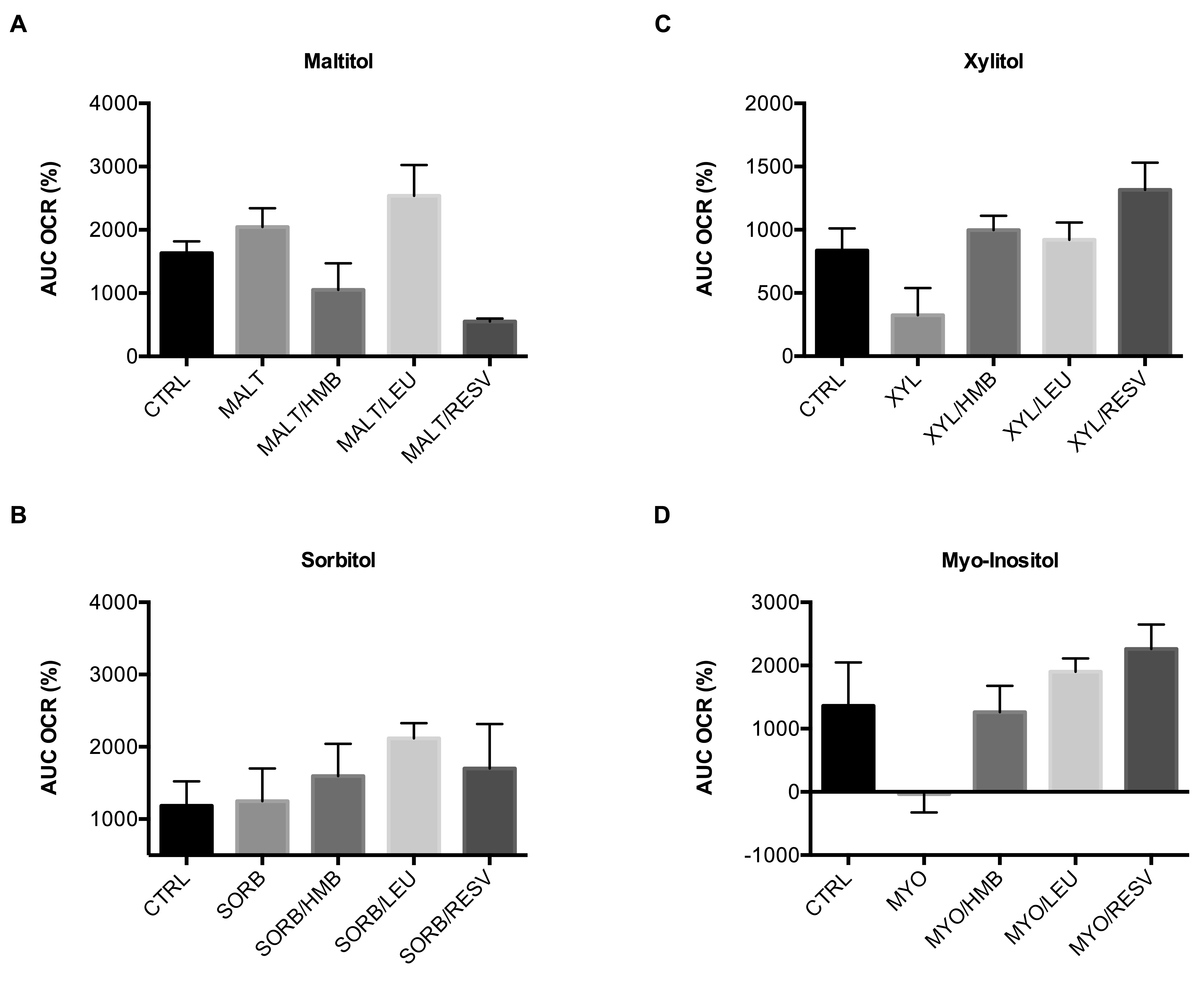

Supplement: Figure S4 — Effects of Leu, HMB or Resv combined with polyols on fatty acid oxidation in C2C12 muscle cells. Differentiated cells were treated with indicated treatments for 24 h. Oxygen consumption rate (OCR) was measured after 200 µM palmitate injection. Effects of combinations of Leu, HMB or Resv with (a) Maltitol (MALT, 0.1 µM), (b) Sorbitol (SORB, 0.5 µM), (c) Xylitol (Xyl, 10 nM) and (d) myo-Inositol (MYO, 0.1 µM) on OCR C2C12 muscle cells. Data are represented as mean ± SEM (n = 4) of calculated areas under the curve (AUC) of OCR in % change from baseline at a two-hour measurement point. *indicates significant difference to control (p≤0.05). (TIFF) [file pone.0089166.s004.tif]

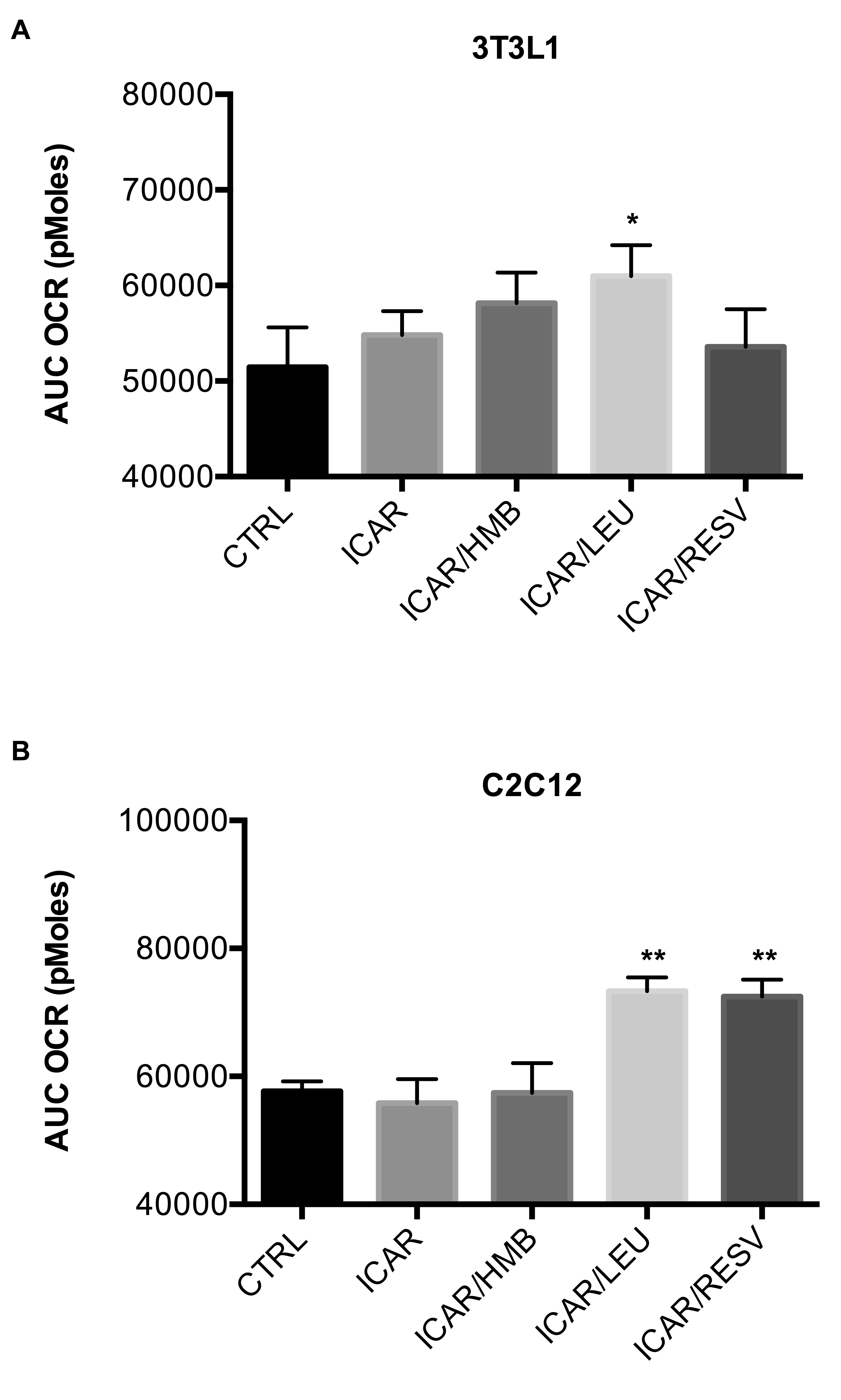

Supplement: Figure S5 — Synergistic effects of Icariin on fatty acid oxidation in 3T3L1 adipocytes and C2C12 muscle cells. Differentiated cells were treated with indicated treatments for 24 h. Oxygen consumption rate (OCR) was measured after 200 µM palmitate injection. Effects of combinations of Leu, HMB or Resv with Icariin (Icar, 1 nM) on OCR in (a) 3T3L1 adipocytes and (b) C2C12 muscle cells. Data are represented as mean ± SEM (n = 4) of calculated areas under the curve (AUC) of OCR in pMoles at a two-hour measurement point. *indicates significant difference to control, **indicates significant difference to control and icariin (p≤0.05). (TIFF) [file pone.0089166.s005.tif]

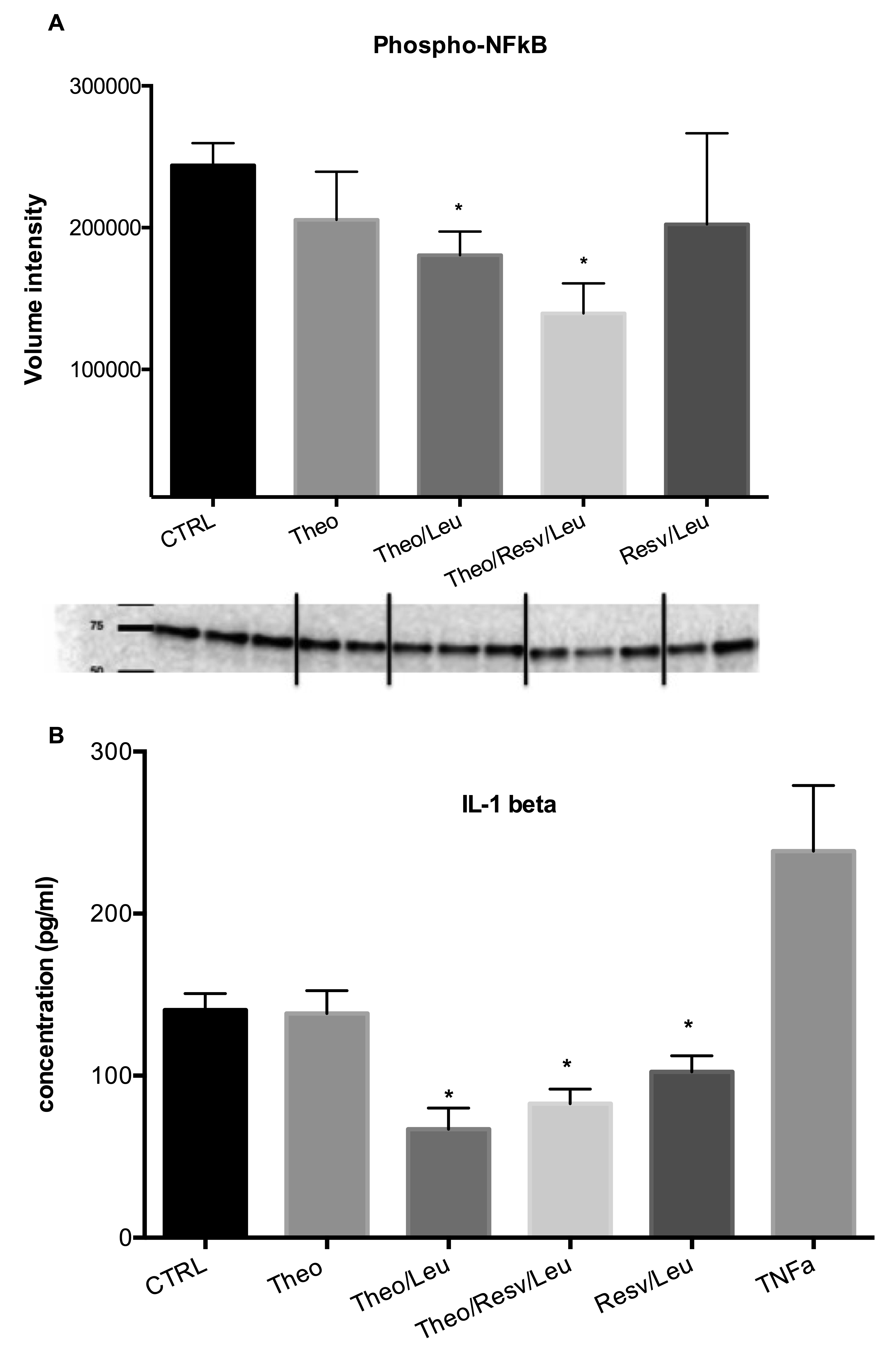

Supplement: Figure S6 — Effects of theophylline-Resv-Leu-combinations on inflammatory biomarker in mouse lung endothelial cells. Mouse lung endothelial cells were treated with 1 µM theophylline alone or in combination with leucine and resveratrol for 24 h. TNF-α (10 ng/ml) was used as positive control. (a) Phospho-NF-κB-expression was determined by Western blot in cell lysate using anti-Phospho-NF-κB antibody (Cell Signaling, Billerica, MA, USA) and bands were quantified using Image Lab Software (Bio-Rad, Hercules, CA, USA). (b) IL-1β release in cell culture media determined via IL-1β ELISA kit (Abcam, Cambridge, MA, USA). Data are represented as mean ± SEM (n = 2 to 4). *indicates significant difference to control (p<0.05). (TIFF) [file pone.0089166.s006.tif]

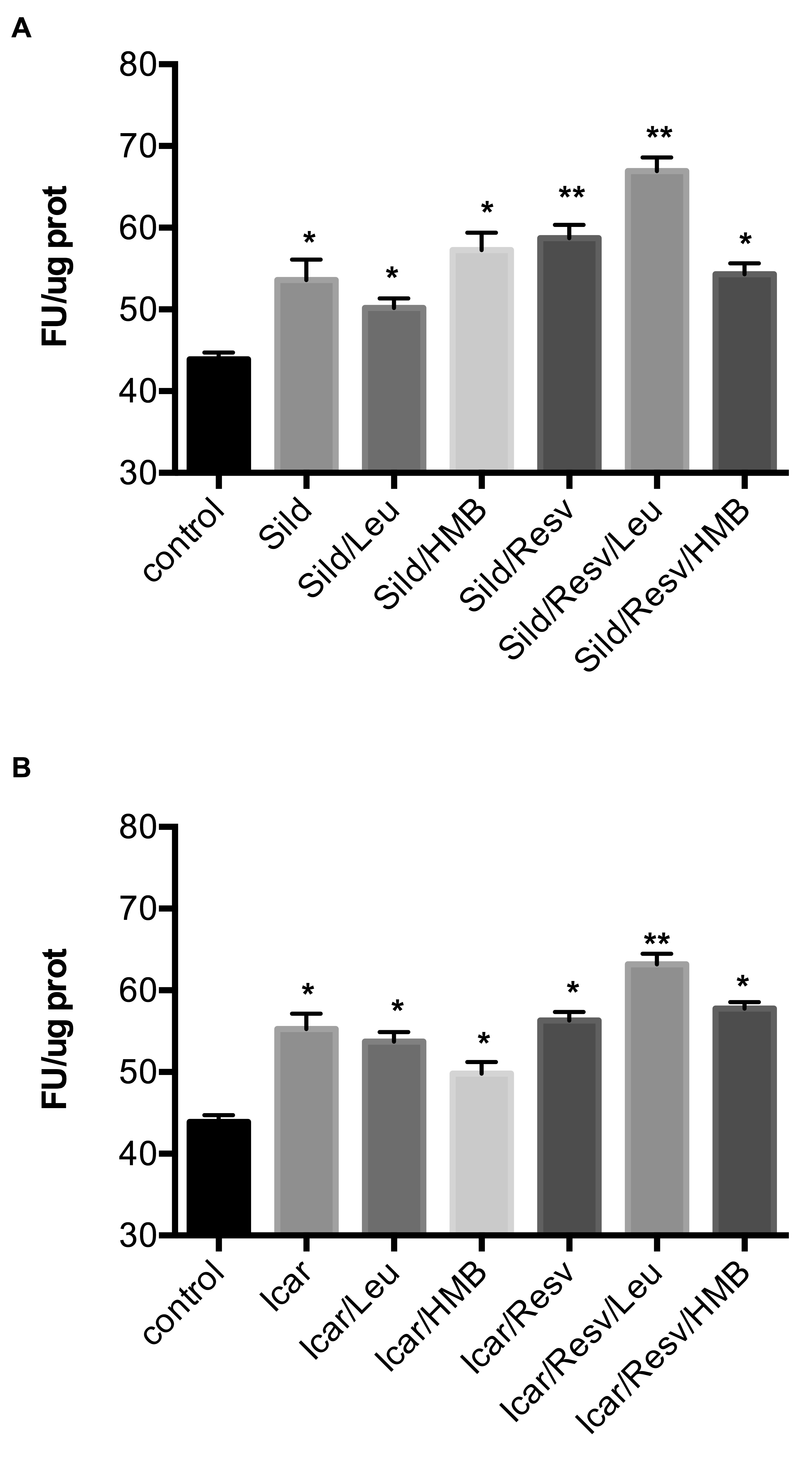

Supplement: Figure S7 — Synergistic effects of Sildenafil and Icariin on nitric oxide (NO) production in C2C12 muscle cells. Differentiated cells were treated with indicated treatments for 4 h. NO production was detected by fluorescence using the fluorophore diaminofluorescein diacetate DAF-2DA (Cell Technology, Inc.). (a) Effects of Sildenafil (Sild, 1 nM) and (b) of Icariin (Icar, 1 nM) combinations with Leu, HMB or Resv in C2C12 muscle cells. Data are represented as mean ±SEM (n = 6). *indicates significant difference to control (p≤0.05), **indicates significant difference to control and Sild (a), or control and Icariin (b) (p≤0.05). (TIFF) [file pone.0089166.s007.tif]
